# Supplementary material for: In vivo optochemical control of cell contractility at single‐cell resolution
Source: EMBO Rep. 2019 Oct 30;20(12):e47755. doi: 10.15252/embr.201947755 (PMC6893293; doi:10.15252/embr.201947755)
Supplement: Supplementary file 1 — Expanded View Figures PDF [file EMBR-20-e47755-s001.pdf]

Expanded View Figures

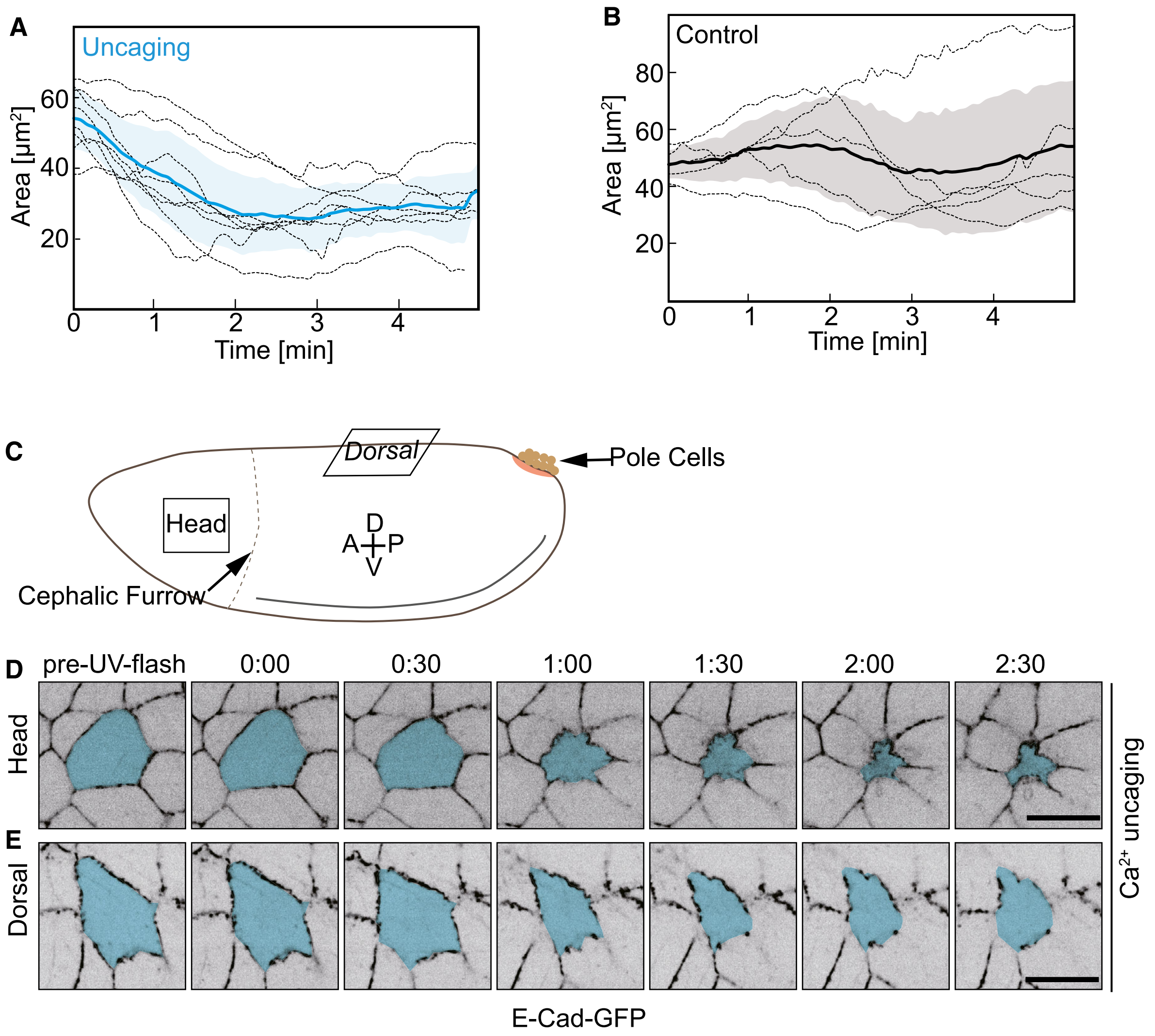

Figure EV1. Cell contraction following CaLM in epithelium.

A, B Cross-sectional cell areas of individual target cells (dashed lines) following a UV laser pulse. (A) Embryos injected with 2 mM NP-EGTA, AM (n = 8 cells in eight embryos). (B) Embryos injected with buffer (n = 5 cells in five embryos). Mean (bold line) with standard deviation of the mean (ribbon).

C Schematic drawing of an embryo shows the head and dorsal region where CaLM was performed.

D, E Images from a time-lapse recording embryos expressing E-Cad-GFP and injected with 2 mM NP-EGTA, AM following with Ca^{2+} uncaging in head (B) or dorsal (C) region. Target cells are labeled in blue.

Data information: Scale bars: 10 µm in (D, E).

Figure EV2. CaLM does not induce apoptosis.

A Images from embryos express α-Catenin-RFP and apoptosensor. Two blue dotted boxes indicate the apoptotic cells. A yellow dotted box indicates the selected cell where CaLM was performed in the same embryos.

B Images of amnioserosa cells from two time-lapse recordings in embryos (stage 14) expressing E-Cad-GFP and injected with 1 mM NP-EGTA, AM followed by UV illumination showing long-term behavior after uncaging. The target cells for CaLM are highlighted in magenta.

C Cross-sectional area of target cells over 30 min after Ca^{2+} uncaging. Cell contraction in 1 out of 3 target cells was reversible in 10 min.

Data information: Scale bars: 10 µm in (A, B).

▶

© 2019 The Authors

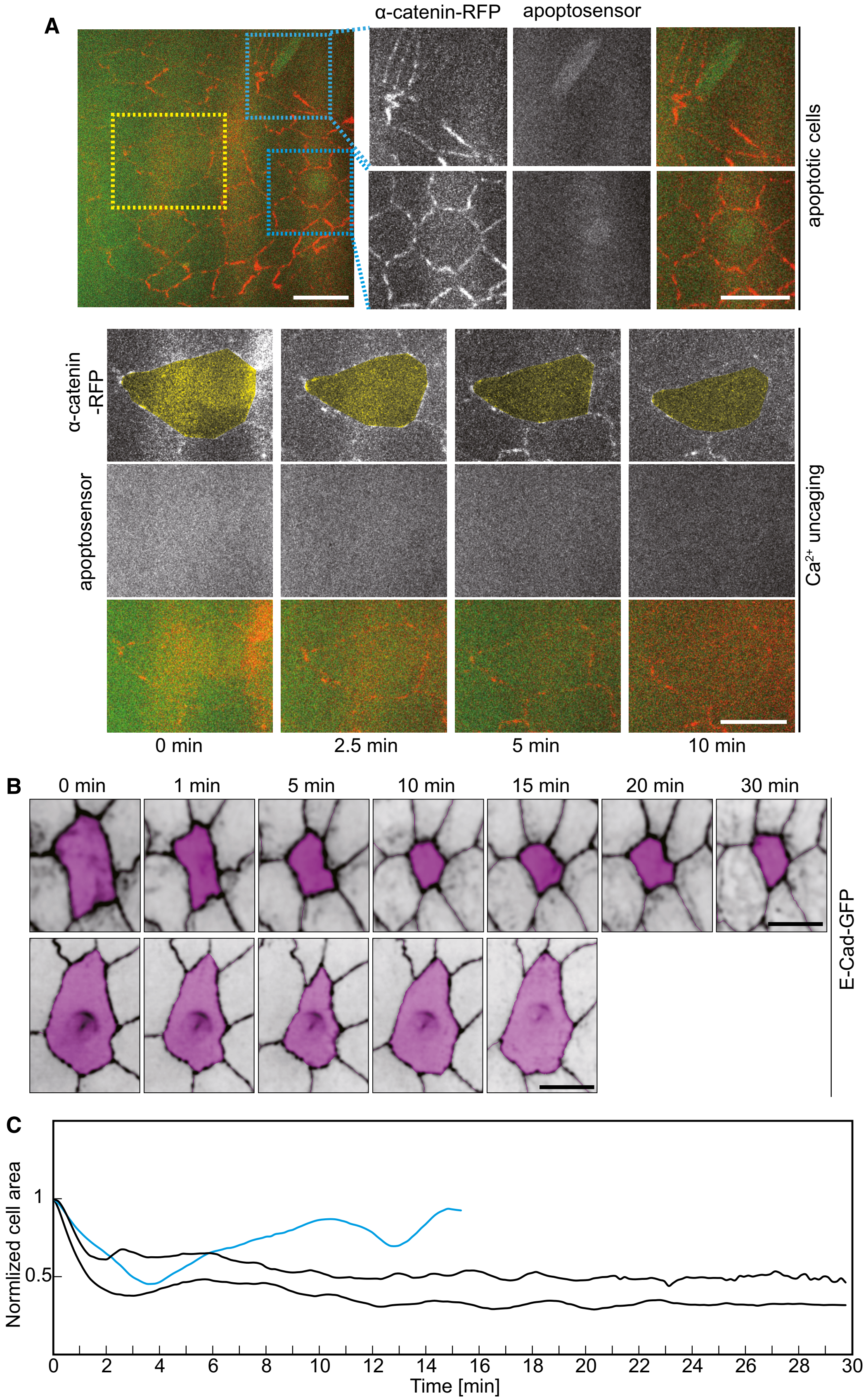

Figure EV2.

 © 2019 The Authors

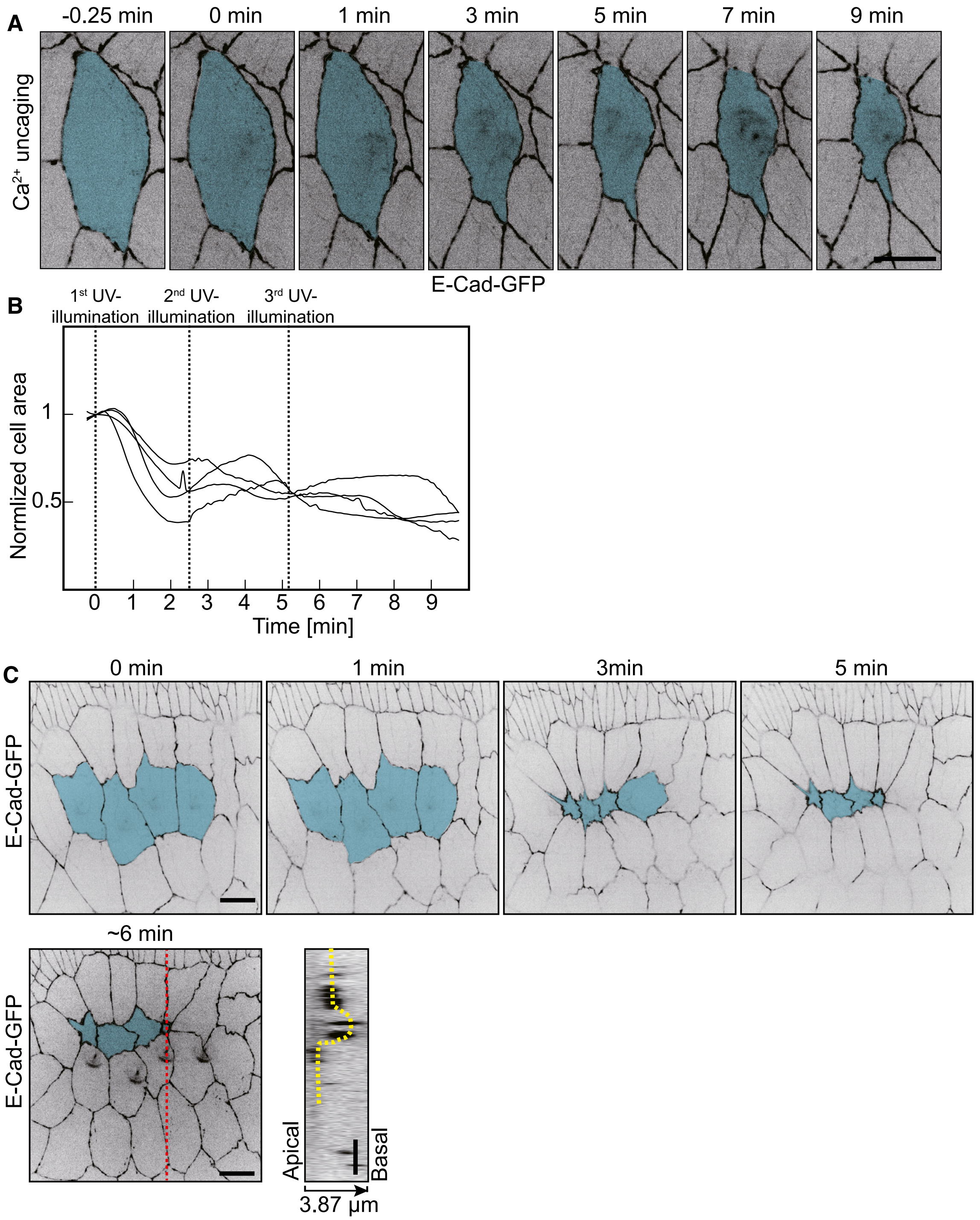

Figure EV3. Cell contraction following CaLM in amnioserosa.

A Images of amnioserosa from time-lapse recording in embryos (stage 14) expressing E-Cad-GFP and injected with 1 mM NP-EGTA, AM following with three times UV illumination (0, 2.5 and 5 min). Target cell is highlighted in blue.

B Cross-sectional cell areas of four individual target amnioserosa cells from the embryos injected with 1 mM NP-EGTA, AM following three times UV laser pulses. Cell area was normalized with the initial size (the first frame of recording after 1st UV illumination). The time points of UV laser pulses are indicated.

C CaLM triggers multiple cell constriction simultaneously in amnioserosa. Images of amnioserosa from a time-lapse recording in embryos (stage 14) expressing E-Cad-GFP and injected with 1 mM NP-EGTA, AM following with UV illumination. The target cells are highlighted in blue. An orthogonal view shows an invagination (yellow dash line) is induced by Ca^{2+} uncaging triggered contraction. The red dash line indicates the region of orthogonal view.

Data information: Scale bars: 10 µm in (A, C).

© 2019 The Authors

Figure EV4. VinculinD1 reporter, Rock inhibitor, *RhoGEF2* mutant, and Rho sensor.

A Scheme of the domain structure of Vinculin. Numbers indicate position of amino acid residues. Transgenic construct with the D1 domain (blue) fused to GFP and expressed under GAL4/UAS control.

B VinculinD1-GFP fluorescence on cell junctions of target and control cells (n = 6 target cells and 6 control cell borders). Mean (bold line) with standard deviation of the mean (ribbon band).

C Scatter plot of normalized cross-sectional area with normalized VinculinD1-GFP intensity in target cells.

D The Rock inhibitor inhibits Ca^{2+}-induced constriction. Cross-sectional areas of eight individual target cells following CaLM in Y-27632 co-injected embryos.

E A confocal cross-sectional image from a *RhoGEF2* germline clone embryo expressing E-Cadherin-GFP shows the characteristic phenotype of multinucleated cells during cellularization. The red asterisks indicate multinucleated cells.

F Axial image stack of an embryo during cellularization showing the functionality of the Rho sensor.

G Images from time-lapse recording of an embryos expressing the Rho sensor. Cells in cytokinesis. Stage 8. Lateral epidermis.

Data information: Scale bar: 10 μm in (B, E, F, G).

© 2019 The Authors

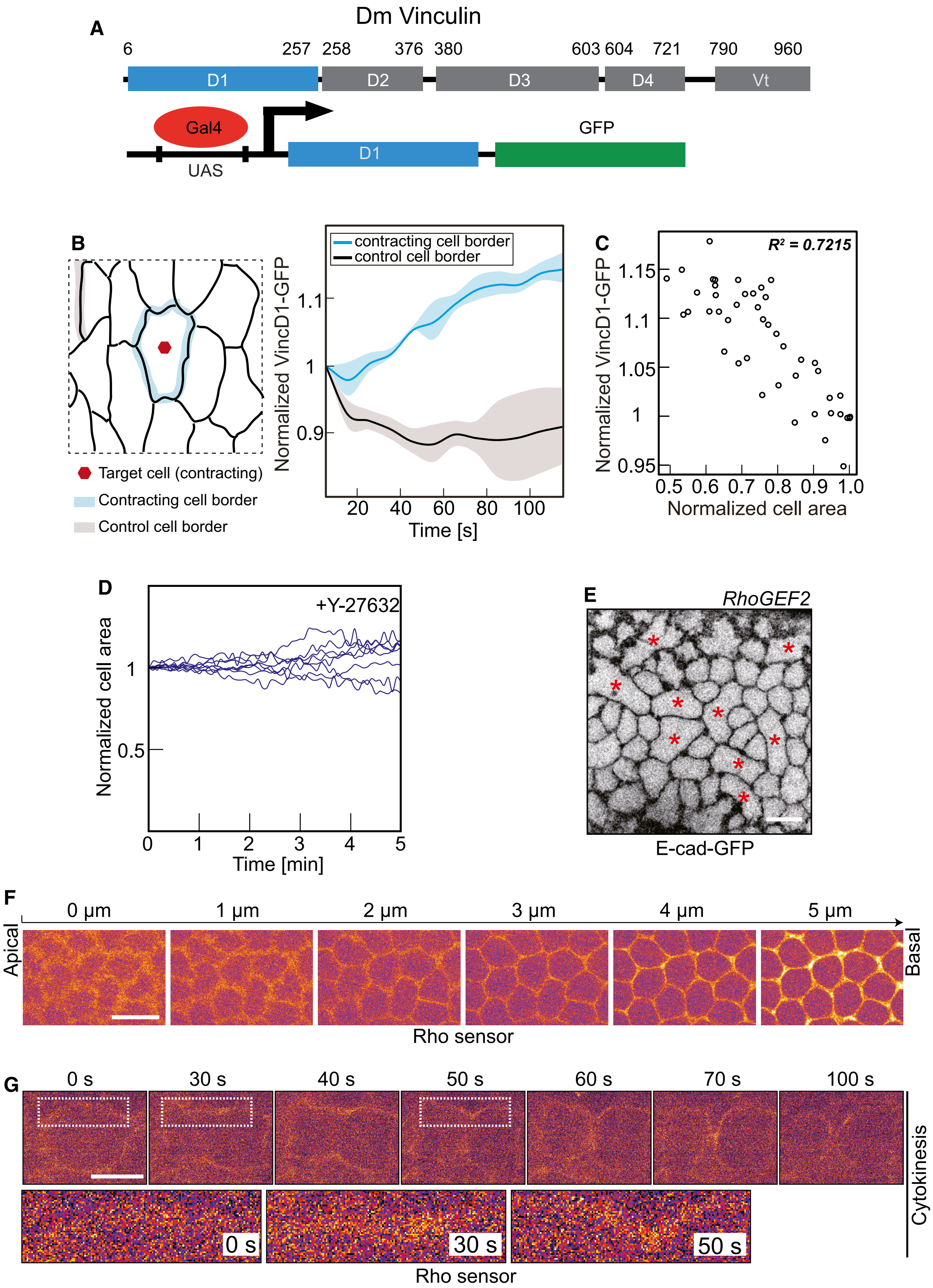
A
Dm Vinculin
6 257 258 376 380 603 604 721 790 960
D1 D2 D3 D4 Vt
Gal4
UAS
D1
GFP
B
Target cell (contracting)
Contracting cell border
Control cell border
contracting cell border
control cell border
Normalized VincD1-GFP
Time [s]
C
R² = 0.7215
Normalized VincD1-GFP
Normalized cell area
D
+Y-27632
Normalized cell area
Time [min]
E
RhoGEF2
E-cad-GFP
F
0 μm 1 μm 2 μm 3 μm 4 μm 5 μm
Apical
Basal
Rho sensor
G
0 s 30 s 40 s 50 s 60 s 70 s 100 s
0 s 30 s 50 s
Cytokinesis
Rho sensor

Figure EV4.

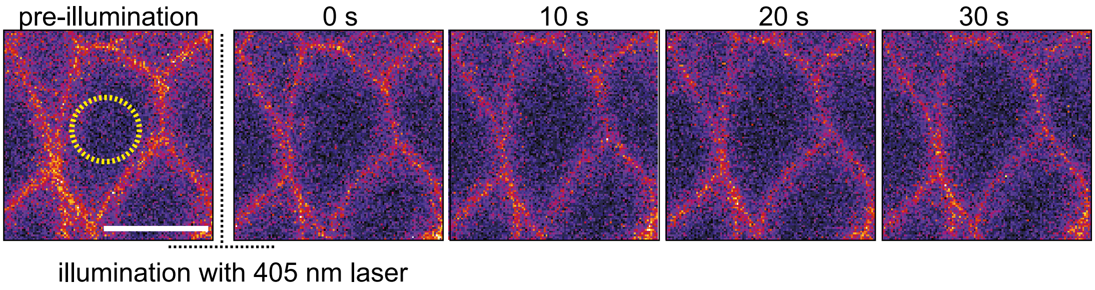

Figure EV5. No increased signal of the GCaMP reporter after illumination by 405-nm laser in single individual cells.
Images from time-lapse recording of embryos (stage 7, lateral epidermis) expressing a membrane-bound Ca^{2+} sensor (GCaMP6-myr) and injected with 2 mM NP-EGTA, AM. Target cells were exposed to a 405-nm cw laser.

Data information: Scale bars: 10 µm.

 © 2019 The Authors
