# Supplementary material for: In vivo optochemical control of cell contractility at single‐cell resolution
Source: EMBO Rep. 2019 Oct 30;20(12):e47755. doi: 10.15252/embr.201947755 (PMC6893293; doi:10.15252/embr.201947755)
Supplement: Supplementary file 2 — Movie EV1 [file EMBR-20-e47755-s002.zip › Movie_EV1.docx]

**Movie EV1 Uncaging induces rapid intracellular Ca^2+^ concentration increasing in epithelial target cells.** Time-lapse recording from embryos expressing UAS-myr-GCaMP6 (lateral epidermis, stage 7). Time in min:sec. Anterior left, dorsal up. This movie relates to Fig 1C, 1F and 6C.
