# Supplementary material for: In vivo optochemical control of cell contractility at single‐cell resolution
Source: EMBO Rep. 2019 Oct 30;20(12):e47755. doi: 10.15252/embr.201947755 (PMC6893293; doi:10.15252/embr.201947755)
Supplement: Supplementary file 3 — Movie EV2 [file EMBR-20-e47755-s003.zip › Movie_EV2.docx]

**Movie EV2 CaLM induces constriction in a columnar epithelium (including the process of uncaging, using CCD camera).** Time-lapse recording from embryos expressing E-Cad-GFP (lateral epidermis, stage 7) recorded with a CCD. The target cell is marked by a red dot. A stack was acquired every 5 seconds. Time in min:sec. Anterior left, dorsal up.
