# Supplementary material for: In vivo optochemical control of cell contractility at single‐cell resolution
Source: EMBO Rep. 2019 Oct 30;20(12):e47755. doi: 10.15252/embr.201947755 (PMC6893293; doi:10.15252/embr.201947755)
Supplement: Supplementary file 4 — Movie EV3 [file EMBR-20-e47755-s004.zip › Movie_EV3.docx]

**Movie EV3 CaLM induces constriction in a columnar epithelium (without the process of uncaging, using emCCD camera).** Time-lapse recording from embryos were expressing E-Cad-GFP recorded with a emCCD. The target cell is marked by a red dot. Stacks were acquired every 5 seconds. Time in min:sec. Anterior left, dorsal up. This movie relates to Fig 2C.
