# Supplementary material for: In vivo optochemical control of cell contractility at single‐cell resolution
Source: EMBO Rep. 2019 Oct 30;20(12):e47755. doi: 10.15252/embr.201947755 (PMC6893293; doi:10.15252/embr.201947755)
Supplement: Supplementary file 5 — Movie EV4 [file EMBR-20-e47755-s005.zip › Movie_EV4.docx]

**Movie EV4 CaLM triggers apical-constriction in a squamous epithelium.** Time-lapse recording of embryos expressing E-Cad-GFP (amnioserosa, stage 14).Stacks were acquired every 5 seconds. Time in min:sec. Anterior left. This movie relates to Fig 3D.
