# Supplementary material for: In vivo optochemical control of cell contractility at single‐cell resolution
Source: EMBO Rep. 2019 Oct 30;20(12):e47755. doi: 10.15252/embr.201947755 (PMC6893293; doi:10.15252/embr.201947755)
Supplement: Supplementary file 6 — Movie EV5 [file EMBR-20-e47755-s006.zip › Movie_EV5.docx]

**Movie EV5 VinculinD1-GFP accumulation following CaLM.** Time-lapse recording from amnioserosa cells in stage 14 embryos were expressing E-Cad-mTomato and VinculinD1-GFP. Time in min:sec. Anterior left. This movie relates to Fig 5B.
