# Supplementary material for: In vivo optochemical control of cell contractility at single‐cell resolution
Source: EMBO Rep. 2019 Oct 30;20(12):e47755. doi: 10.15252/embr.201947755 (PMC6893293; doi:10.15252/embr.201947755)
Supplement: Supplementary file 7 — Movie EV6 [file EMBR-20-e47755-s007.zip › Movie_EV6.docx]

**Movie EV6 Y-27632 does not disturb Ca^2+^ uncaging.** Time lapse recording from embryos expressing UAS-myr-GCaMP6 and injected with 10 mM Y-27632 and 2 mM NP-EGTA,AM. A stack was acquired every second. Time in min:sec. Anterior left, dorsal up. This movie relates to Fig 6C.
