# Supplementary material for: In vivo optochemical control of cell contractility at single‐cell resolution
Source: EMBO Rep. 2019 Oct 30;20(12):e47755. doi: 10.15252/embr.201947755 (PMC6893293; doi:10.15252/embr.201947755)
Supplement: Supplementary file 8 — Movie EV7 [file EMBR-20-e47755-s008.zip › Movie_EV7.docx]

**Movie EV7 CaLM fails to trigger apical-constriction in Y-27632 injected embryos.** Time lapse recording from embryos were expressing E-Cad-GFP and co-injected with 10 mM Y-27632 and 2 mM NP-EGTA, AM (lateral epidermis, stage 7). The target cell is marked by a red dot. Time in min:sec. Anterior left, dorsal up. This movie relates to Fig 6E.
