# Supplementary material for: In vivo optochemical control of cell contractility at single‐cell resolution
Source: EMBO Rep. 2019 Oct 30;20(12):e47755. doi: 10.15252/embr.201947755 (PMC6893293; doi:10.15252/embr.201947755)
Supplement: Supplementary file 9 — Movie EV8 [file EMBR-20-e47755-s009.zip › Movie_EV8.docx]

**Movie EV8 *RhoGEF2* is not required for CaLM induced constriction.** Time lapse recording from embryos with *RhoGEF2* germline clones expressing E-Cad-GFP (lateral epidermis, stage 7). Time in min:sec. Anterior left, dorsal up. This movie relates to Fig 6F.
