# Supplementary material for: In vivo optochemical control of cell contractility at single‐cell resolution
Source: EMBO Rep. 2019 Oct 30;20(12):e47755. doi: 10.15252/embr.201947755 (PMC6893293; doi:10.15252/embr.201947755)
Supplement: Supplementary file 10 — Movie EV9 [file EMBR-20-e47755-s010.zip › Movie_EV9.docx]

**Movie EV9 Rho A sensor activity is not induced by CaLM.** Time-lapse recording from embryos were expressing a Rho sensor (lateral epidermis, stage 7). The target cell is marked by a blue dot. Time in min:sec. Anterior left, dorsal up. This movie relates to Fig 6G.
